# Introducing Osteopathic Curriculum for Family Medicine Physicians in a Community-based Allopathic Residency Program

**DOI:** 10.51894/001c.9059

**Published:** 2019-07-01

**Authors:** Jacob Turnbull, Dan Merck, Karri MacMillan

**Affiliations:** 1 Family Medicine Mercy Health Saint Mary’s; 2 Medical Student Michigan State University College of Osteopathic Medicine; 3 Family Medicine Mercy Health St. Mary’s

**Keywords:** allopathic family medicine residency, osteopathic manipulative treatment (omt), osteopathic curriculum

## Abstract

**CONTEXT:**

Will increased exposure to osteopathic medicine improve allopathic resident and attending physicians’ support of implementing an osteopathic curriculum in graduate medical education? The overall goal of this quality improvement project was to examine the familiarity and interest of allopathically-trained residents and attendings with osteopathic medicine before, and after, a brief educational workshop.

**METHODS:**

Setting: Mercy Health Saint Mary’s Hospital in Grand Rapids, MI. Participants included a sample of 27 Family Medicine (FM) residents and attendings who had been trained in allopathic medical schools. First, a one-hour lecture on “Foundations of Osteopathic Medicine” was given. The lecture included a PowerPoint presentation followed by a 15-minute hands-on demonstration of osteopathic diagnosis and treatment. A pre and post-workshop survey modified from another larger study was administered to all attendees. Primary selected outcomes included percentage of allopathic resident participation in attendance and pre-post-workshop response differences.

**RESULTS:**

Of the 31 allopathic residents in this community-based FM program, 23 (74.2%) were present for the lecture and completed both a pre and post-workshop survey. A total of 24 (78%) of participants had never attended a previous lecture on osteopathic medicine. Respondents’ overall attitude regarding the implementation of an osteopathic curriculum was generally positive after the workshop. Of the 27 participants (23 residents and four attendings) surveyed before the lecture, 23 (85.2%) were initially interested in learning how to perform osteopathic manual treatment (OMT), this increasing to 25 (92.6%) after the workshop. The Mercy Health FM resident respondents were initially first supportive of osteopathic medicine at 59.3%, improving to 77.8% after completing the workshop.

**CONCLUSIONS:**

These results suggest that many allopathically trained physicians may lack exposure to osteopathic medicine principles. In this allopathic-oriented residency sample setting, a relatively brief workshop increased attendees’ interest in osteopathic medicine. Results suggest that initial and refresher exposure to osteopathic medicine content and OMT practices during residency curricula can be used to elicit support from allopathically-trained resident physicians.

## INTRODUCTION

By 2020, the merger of the Accreditation Council for Graduate Medical Education (ACGME) and the American Osteopathic Association (AOA) will fully integrate the accreditation of both allopathic and osteopathic residency programs. An all-time high of 3,590 doctors of osteopathy (DO) applied in the 2017 allopathic (MD) residency matching program.^1^

During that same year, 17.85% of applicants who matched to PGY-1 Family Medicine (FM) positions were DO.^2^ The expectation is that the number of DO residents training in MD-oriented residencies will continue to increase with the merger.^3,4^ It is now considered increasingly important that at least some osteopathic medicine content is integrated into more graduate medical education (GME) curricula.^4,5^

Osteopathic recognition is offered to programs to increase the osteopathic curriculum for DO residents in MD-oriented programs.^3^ In 2015, Busey et al. successfully implemented an osteopathic curriculum in a military allopathic residency, although the effects on allopathic (MD) colleagues was unknown since only DO physicians were surveyed.^4^ In 2005, Allee et al. surveyed a sample of 232 MD residents and found that 173 (74.6%) of respondents reported having received no prior exposure to osteopathic manipulative treatment (OMT) techniques during medical school. However, 165 (71.1%) of surveyed MD were still interested in learning more about osteopathic medicine.^5^

Since its inception in 1892 by Andrew Taylor Still in Kirksville, Missouri, osteopathic education has retained a distinct identity from allopathic medicine.^6^ Over the years, several historical events have configured the major elements of osteopathic medical education to allopathic norms including curriculum changes prompted by the Flexner report in 1910, the 1929 addition of pharmacology content, increasing undergraduate requirements in 1940, and equivalent licensing privileges as a result of the California Merger of 1961.^7,8^

As the number of DO medical schools increased for a limited set of DO-oriented residency program slots, the AOA allowed DO graduates the option of completing MD-oriented residency programs.^9^ This resulted in nearly 60% of DO graduates enrolled in MD-oriented programs by 1995. This was concluded by experts to impose a challenge to maintaining the distinct identity of osteopathic medicine and practices.^9^

In 1998, Dr. Johnson and colleagues suggested that osteopathic medicine educational didactics be offered for MD physicians during residency, and stressed the importance of appointing more DO faculty.^10^ In 2005, Leiber and colleagues demonstrated that MD residents could learn osteopathic principles and obtain basic OMT skills during a one-month elective. The OMT elective included eight to 12 half-day OMT clinic sessions. The course included patient encounters, one-on-one instruction with DO attendings, interactive CD-ROM instruction and assigned readings. After the elective, all allopathic residents received a post-test and were found to be overall proficient in defined OMT skills.^11^

In 2008, another MD-oriented program that was unable to become dually accredited developed an osteopathic curriculum and OMT clinic to develop residents’ skills.^12^ This approach was effectively repeated in an MD-oriented military residency program in 2015 at a resident-led OMT clinic.^4^ Both Rubeor et al.^12^ and Busey et al.^4^ utilized a pre-survey to better understand the resident’s perspectives of the current osteopathic curriculum. In each setting, an OMT clinic was established as the intervention. Rubeor et al. measured AOA recognition of residents’ osteopathic intern year as their primary outcome while Busey et al. repeated their initial survey after the implementation of their new OMT clinic. The generalizability of these results to other settings is limited since their sample sizes were small (i.e., three and nine residents respectively) and only DO residents were included.^4,12^

A 2016 survey indicated that Osteopathic Recognition and OMT refresher courses during residency programs remains an important factor for DO medical students.^13^ In another recent survey, 497 (68%) of third year DO medical student respondents stated that a residency program with Osteopathic Recognition would increase its appeal.^14^

Still, there has apparently been no recently published survey projects gauging the interest of MD-trained residents or attendings in learning osteopathic medicine and OMT principles. It was therefore the overall goal of this quality improvement project to survey the familiarity and interest in osteopathic medicine from a sample of MD residents and attendings before and after participating in a brief GME workshop.

### Project Objectives

The primary objectives were to: 1. Determine the familiarity and exposure of MD physicians with osteopathic medicine in a community-based MD-oriented FM residency program, 2. Evaluate MD residents’ attitudes towards osteopathic medicine before and after participating in an OMT educational workshop, and 3. To increase knowledge and awareness of the possible indications of OMT in FM for physician participants.

## METHODS

Since this project was conducted as a quality improvement Initiative at Mercy Health, it was ruled to be exempt from formal review by the Mercy Health Regional Institutional Review Board.

### Pre- and Post-Workshop Surveys

An unvalidated pre and post-workshop survey similar to Allee et al.^5^ was developed and approved by residency program leadership. (Figure 1 and Figure 2) The pre-survey included two questions regarding demographics pertaining to the participant's current level of training in the health system and medical school in which they graduated. The pre-survey also contained two questions related to previous osteopathic medicine familiarity and exposure. Both the unvalidated pre and post-workshop survey contained seven identical questions which would be used for pre-post inferential statistical comparisons (Figures 1 and 2).

Each pair of participant surveys was also initially assigned a unique number, so that potential differences between the pre- and post-workshop responses could be compared. There were no other identifiers on the survey, so there was no way to link a specific individual with a specific set of survey responses.

### OMT Workshop

The OMT workshop for this project included a one-hour PowerPoint lecture entitled “Foundations of Osteopathic Medicine” which was delivered as a grand rounds lecture. The content of the workshop primarily introduced attendees to the four tenets of osteopathic medicine:

1. The human being as a dynamic unit of function
2. The body possesses self-regulatory mechanisms that are self-healing in nature
3. Structure and function are interrelated on all levels
4. Rational treatment is based on these principles ^6^

The lecture was followed by 15-minute hands-on demonstration of OMT techniques which included counterstain, myofascial release, muscle energy and high velocity low amplitude OMT treatments. All residents and attending participants were members of the ACGME-accredited Mercy Health Grand Rapids FM Residency program located in Grand Rapids, Michigan.

Prior to the OMT workshop, resident and attending physician attendees completed the 20-item survey questionnaire asking about their ratings concerning dimensions of OMT training for allopathically-trained physicians. To examine for pre-post-workshop differences, attendees were asked after the workshop to complete a largely-identical survey questionnaire. Survey items were on a three or four-category Likert-type scale that was later conservatively collapsed into equivalent-sized numerical categories. (Figures 1 and 2).

Preliminary descriptive statistical analyses confirmed that the distribution of survey item opinion responses were non-normal (i.e., non-parametric) in nature as might be easily expected from a smaller respondent sample in a single FM residency program. In response, a series of non-parametric Wilcoxon Matched Pair Signed Rank t test analytic procedures^15^ were conducted by the Michigan State University Statewide Campus System using SPSS Version 25 analytic software.^16^ These analytic procedures are particularly suitable for smaller non-parametric samples and were used to test for possible statistically significant pre-post-workshop differences in individual resident opinion ratings.^15^

## RESULTS

Of the 31 allopathic residents in the FM program, 27 (74.2%) were present for the lecture and completed both a pre and post-workshop survey. The training levels of the sample was diverse with seven (26%) being PGY-1 residents, six (22%) PGY-2 residents, 10 (37%) PGY-3 residents and four (15%) attending physicians completing surveys. The majority, (I e., 19 (70.4%) had completed medical school in the United States. (Table 1)

**Table 1:** Demographics

**PGY**	**N = 27**
PGY-1	7 (25.9%)
PGY-2	6 (22.2%)
PGY-3	10 (37.0%)
Attending	4 (14.8%)
**Medical school grad**	
United States	19 (70.4%)
Foreign	8 (29.6%)

Data concerning respondents’ level of familiarity with OMT procedures, as well as varied OMT exposures were also collected. Only one (3.7%) respondent indicated that they were “very familiar” with OMT before the workshop, leaving 13 (48.1%) “somewhat familiar” and 13 (48.1%) “unfamiliar.” Reported types of previous OMT exposure also varied, as 16 (59.3%) had experience through demonstrations, three (11%) through reading, seven (26%) had personal experience, five (18.5%) had exposure through a fellow resident. Only six (22.22%) respondents reported having ever received a past OMT lecture and seven (26%) participants reported having never had any previous experience with OMT. (Table 2)

**Table 2:** Prior OMT Familiarity and Exposure

**OMT familiarity**	**N = 27**
Very familiar	1 (3.6%)
Somewhat familiar	13 (48.1%)
Unfamiliar	13 (48.1%)
**Prior OMT exposure**	
lectures	6 (22.2%)
demonstrations	16 (59.3%)
reading	3 (11.1%)
personal experience	7 (25.9%)
fellow resident	5 (18.5%)
cont. med ed (CME)	0 (0%)
None	7 (25.9%)

Respondents’ overall attitudes regarding the implementation of an OMT curriculum were also positive and increased significantly after the “Foundations of Osteopathic Medicine” workshop was completed. (p = 0.001) Of the 27 residents and attendings who were surveyed before the workshop, 23 (85.2%) were interested in learning how to perform OMT, increasing slightly to 25 (92.6%) after completing the workshop. (p = 0.560) (Tables 3 and 4)

**Table 3:** Pre and Post OMT Workshop Survey Results

**Survey Questions:**	**Pre**	**Post**
***1. To what extent do you feel OMT is effective for somatic dysfunction?***	**(N = 27)**	**(N = 27)**
Very effective	4 (14.8%)	10 (37.0%)
Somewhat effective	22 (81.5%)	17 (62.9%)
Not effective	1 (3.7%)	N/A
***2. To what extent do you feel OMT is effective for systemic illness (e.g., asthma)?***		
Very effective	2 (7.4%)	2 (7.4%)
Somewhat effective	9 (33.3%)	21 (77.8%)
Not effective	16 (59.3%)	4 (14.8%)
***3. To what extent are you interested in learning how to perform OMT?***		
Very interested	11 (40.7%)	15 (55.6%)
Somewhat interested	12 (44.4%)	10 (37.0%)
Not interested	4 (14.8%)	2 (7.4%)
***4. Should OMT be incorporated into allopathic medical school curricula?***		
Strongly support	4 (14.8%)	12 (44.4%)
Support	13 (48.1%)	10 (37.0%)
Neutral	10 (37.0%)	4 (14.8%)
Oppose	N/A	1 (3.7%)
***5. Should OMT be incorporated into allopathic residency program curricula?***		
Strongly support	6 (22.2%)	13 (48.1%)
Support	10 (37.0%)	8 (29.6%)
Neutral	10 (37.0%)	5 (18.5%)
Oppose	1 (3.7%)	1 (3.7%)
***6. Would you be interested in more OMT CME geared towards teaching MDs?***		
Very interested	9 (33.3%)	17 (62.9%)
Somewhat interested	16 (59.3%)	9 (33.3%)
Not interested	2 (7.4%)	1 (3.7%)
***7. Do you feel the American Osteopathic Association should provide certification for MDS who have tested proficient in the use of OMT?***		
Strongly support	14 (51.8%)	17 (62.9%)
Support	8 (29.6%)	7 (25.9%)
Neutral	4 (14.8%)	2 (7.4%)
Oppose	1 (3.7%)	1 (3.7%)

Using the selected analytic software, each of seven key paired pre-post-workshop survey ratings levels were shown to have numerically increased, with four items reaching statistical significance (I e., p value of less than 0.05). (Table 4)

**Table 4:** Pre and Post-OMT Workshop Scores

**Individual OMT Item Scores (range 0 -3 or 0-2)**	**Pre-Workshop Mean Score (N = 27)**	**Post-Workshop Mean Score (N = 27)**	**Difference In Pre-Post Means**	**t**	**Df**	**p-value**
1. “To What Extent do You Feel OMT is Effective for Somatic Dysfunction?” (possible three-point scale)	1.11 (SD 0.424)	1.37 (SD 0.492)	+ 0.26	-3.017	26	0.006 **
2. “To What Extent do You Feel OMT is Effective for Systemic Illness”	0.48 (SD 0.643)	0.93 (SD 0.474)	+0.45	-4.561	26	**< 0.001**
3. “To What Extent are You Interested in Learning How to Perform OMT?”	1.26 (SD 0.712)	1.48 (SD 0.643)	+0.22	-2.000	26	0.56
4. “Should OMT be Incorporated into Allopathic Medical School Curricula?”	1.78 (SD 0.698)	2.22 (SD 0.847)	+0.44	-4.000	26	**< 0.001**
5. “Should OMT be Incorporated into Allopathic Residency Program Curricula?”	1.78 (SD 0.847)	2.22 (SD 0.892)	+0.44	-3.606	26	**0.001**
6. “Would You Be Interested in More OMT CME geared toward Teaching MDs?”	1.26 (SD 0.594)	2.33 (SD 3.772)	+1.07	-1.392	26	0.176
7. “Do You Feel the AOA should Provide Certification for MDs who have Tested Proficient in Use of OMT?”	2.30 (SD 0.869)	2.48 (SD 0.802)	+0.18	-1.727	26	0.096

* Series of Wilcoxon Matched Pair Signed-Rank t Tests

** Statistically Significant Differences at Alpha of less than 0.05 are listed in Bold font

## DISCUSSION

The results from this study demonstrate that most respondents in this MD-oriented FM residency program had not been very familiar with osteopathic medicine principles or techniques.

Table 2 demonstrated that after receiving the “Foundations of Osteopathic Medicine” lecture the overall attitudes toward osteopathic medicine improved with 25 (93%) respondents being interested in learning more about OMT in the future.

Following the workshop, no respondents believed that OMT was “not effective” in the treatment of somatic conditions. Both the questions pertaining to somatic and systemic effectiveness of OMT had statistically significant pre-post-workshop increases with p values of 0.006 and <0.001 respectively. (Table 4) This may be due to the fact that workshop lecturers had repeatedly emphasized that “form” and “function” were related, and that systemic disease could can benefit from somatic OMT treatments.

Before the workshop, our respondents were generally supportive of implementing an osteopathic curriculum content into MD-oriented programs, with 16 (59.3%) choosing either “strongly support” or “support”, 10 (37%) “neutral” and only one (3.7%) opposed. (Table 3) When asked if the AOA should provide certification for MDs who have tested proficient in the use of OMT, 14 (51.8%) MDs in our program chose “strongly support” despite having very little prior exposure to OMT. (Table 3)

With more MDs presumably increasingly interested in learning OMT, hands-on training and refresher experiences similar to Lieber, et al. (2005)^11^ could be considered for both MD and DO resident trainees. Perhaps those MDs who demonstrate competency with OMT in the future could obtain some type of recognized certification.^11^

It is notable that changes in three survey items (i.e., Questions 3 , 6 and 7 in Table 4) did not increase at statistically significant levels. These survey items could be interpreted as requiring a more personal action/performance from the respondents or systemic developments from the osteopathic profession than the other values/attitude-type survey items. The delivered content of the OMT workshop, sampling error and our small sample size may have also limited our ability to detect significant pre-post workshop differences from these items.

Question 2 (see Table 3) asked “To what extent are you interested in learning how to perform OMT.” Since our PowerPoint presentation comprised the majority of the OMT workshop, only fifteen minutes was allotted to demonstrating OMT techniques. This short duration of OMT exposure may have not been enough to significantly influence our post-survey item responses. Both Questions 6 and 7 differences were non-significant and may have also been less immediately relevant to the residents who were surveyed.

### Project Limitations

Our ability to detect some pre-post-workshop differences may have been limited by our small sample size of FM respondents. Due to the size of our sample subgroups, resident and attending responses could not be realistically compared during analysis of final survey results.

Although the majority of FM residents (74.2%, n = 23) in this study setting were surveyed, only a minority of eligible attendings participated. Two attendings in this residency program were not surveyed as they had been osteopathically trained and the aim of this project was to measure the opinions of allopathically trained physicians.

### Next Steps

Next steps may involve implementing a lengthier osteopathic curriculum lecture series on “Osteopathic Medicine” for incoming residents and attending faculty. Similar to some earlier cited works,^4,12^ our FM program leadership now aspires to begin an OMT clinic in the Wege Family Medicine Residency Center. However, the Mercy Health Grand Rapids Family Residency currently only has two DO residents, making staffing a prospective OMT clinic more challenging.

## CONCLUSIONS

These results suggest that a relatively brief one-hour workshop may serve to improve some MD physicians’ interest levels toward osteopathic medicine and OMT techniques. However, the upcoming ACGME and AOA merger provides both challenges and opportunities for the future of community-based and other GME settings.^9,14^ Ideally, osteopathic GME curricular offerings can help DO residents maintain their OMT skills and practice them alongside their broader group of interested MD colleagues.

It may be beneficial for the AOA and ACGME to continue to collaborate and clarify how MDs might be able to learn and implement osteopathic medicine principles and techniques in the future. The conduction of larger-scale studies with multiple residency programs, medical students and other clinical specialties may clarify how to best implement such GME training workshops across the nation.

### Conflict of Interest

The authors declare no conflict of interest.

## Figures and Tables

**Figure:**
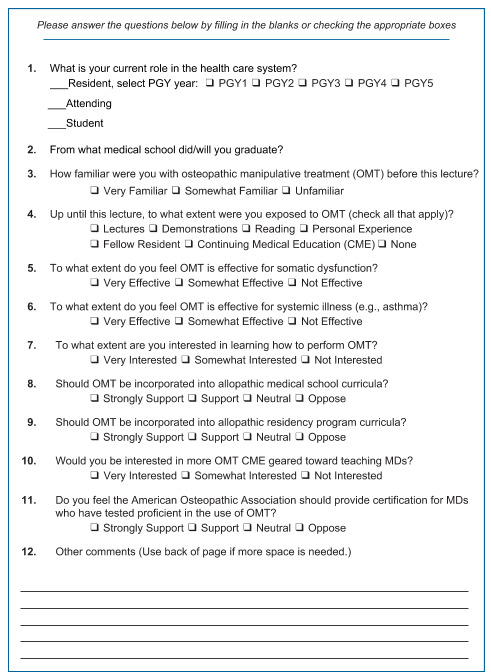
Introducing Osteopathic Curriculum for Residents in an Allopathic Residency – Pre-Workshop Survey Adapted from: Allee BA, Pollak MH, Malnar KF. Survey of osteopathic and allopathic residents’ attitudes toward osteopathic manipulative treatment. J Am Osteopath Assoc. 2005;105(12):551-561.

**Figure:**
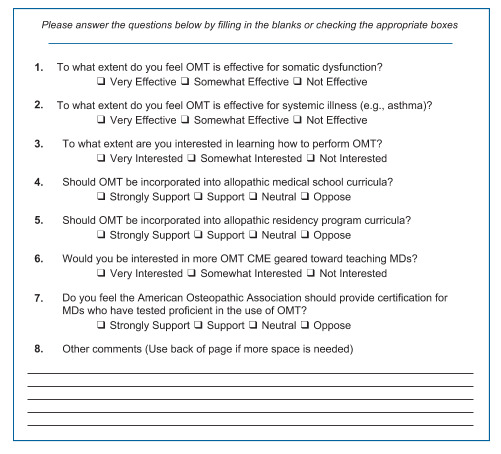
Introducing Osteopathic Curriculum for Residents in an Allopathic Residency – Post-Workshop Survey Adapted from: Allee BA, Pollak MH, Malnar KF. Survey of osteopathic and allopathic residents’ attitudes toward osteopathic manipulative treatment. J Am Osteopath Assoc. 2005;105(12):551-561.
